# Activation of Neuregulin 1/ErbB Signaling Is Involved in the Development of TOCP-Induced Delayed Neuropathy

**DOI:** 10.3389/fnmol.2018.00129

**Published:** 2018-04-23

**Authors:** Hai-Yang Xu, Pan Wang, Ying-Jian Sun, Ming-Yuan Xu, Li Zhu, Yi-Jun Wu

**Affiliations:** ^1^Laboratory of Molecular Toxicology, State Key Laboratory of Integrated Management of Pest Insects and Rodents, Institute of Zoology, Chinese Academy of Sciences, Beijing, China; ^2^Department of Veterinary Medicine and Animal Science, Beijing University of Agriculture, Beijing, China

**Keywords:** organophosphate-induced delayed neuropathy, hen, neuregulin 1, ErbB, lapatinib, neuropathy target esterase, Schwann cell

## Abstract

Organophosphate-induced delayed neuropathy (OPIDN) is characterized by progressive axonal degeneration and demyelination of the spinal cord and sciatic nerves. The neuregulin 1/epidermal growth factor receptor (ErbB) signaling pathway is crucial for axonal myelination. In this study, we investigated whether the neuregulin 1/ErbB signaling pathway mediated the progression of OPIDN. Adult hens were given tri-*o*-cresyl phosphate (TOCP), a typical neuropathic organophosphorus compound, to induce OPIDN. The ErbB inhibitor lapatinib was administered to hens 4 h prior to and 4 days after TOCP exposure. The neuregulin 1/ErbB signaling pathway was examined for their role in maintaining spinal cord and sciatic nerve fiber integrity. Schwann cell line sNF96.2 was used as the *in vitro* cell model. The *in vivo* results showed that TOCP (750 mg/kg body weight, *p.o*.) induced prominent ataxia and significant axon degeneration in the spinal cord and sciatic nerves. Lapatinib (25 mg/kg body weight, *p.o*.) treatment attenuated OPIDN clinically and histopathlogically and partially prevented the TOCP-induced activation of neuregulin 1/ErbB signaling pathway. Lapatinib also prevented the TOCP-induced inhibition of neuropathy target esterase (NTE), a key enzyme during the development of OPIDN, and the disturbed metabolism of phosphatidylcholine in sciatic nerves. In addition, lapatinib was shown, *in vitro*, to protect sNF96.2 cells from TOCP-induced dedifferentiation through neuregulin 1/ErbB signaling. Our results suggest that neuregulin 1/ErbB, through regulation of NTE activity in the peripheral nervous system, mediates the progression of OPIDN. Thus, this signal may serve as a potential target for the treatment of OPIDN.

## Introduction

Organophosphorus compound (OP)-induced delayed neuropathy (OPIDN) is a neurodegenerative disorder characterized by distal degeneration of long and large-diameter axons in the spinal cord and peripheral nerves (Lotti, 1991; Abou-Donia, 1993; Johnson, 1993). The axonal degeneration in OPIDN resembles classical Wallerian degeneration, which is a type of atrophic degeneration that occurs in neurons distal to the site of traumatic axonal injury (Bouldin and Cavanagh, 1979). Clinical manifestations of OPIDN include ataxia, muscle weakness, and subsequent severe hindlimb paralysis (Emerick et al., 2012). Adult hens have been extensively used as an animal model of OPIDN because they are sensitive to OP-induced delayed neuropathy, and their toxicological signs are similar to those seen in humans (Zhu et al., 2016).

A large-scale outbreak of OPIDN occurred in the United States during the 1930s. Drinking “Ginger Jake” contaminated with tri-*o*-cresyl phosphate (TOCP) caused OPIDN in estimated 50,000 people (Morgan and Tulloss, 1976). There were also occurrences of OPIDN in Morocco, Holland, Fiji, Yugoslavia, France, South Africa, Sri Lanka and India. These incidences were caused by consumption of cooking oil contaminated with lubricating oil containing TOCP, which lead to paralysis in thousands of individuals (Nanda and Tapaswi, 1995). Despite the widespread and catastrophic outbreaks of OPIDN, the molecular mechanisms underlying this disorder are still not clear. Neuropathy target esterase (NTE) is a membrane protein located on endoplasmic reticulum. Direct OP-induced inhibition of NTE and subsequent generation of “aged NTE” are prerequisites of OPIDN (Hou et al., 2008). NTE has phospholipase B activity and hydrolyzes phosphatidylcholine in both mammalian cells and yeast (Zaccheo et al., 2004; Glynn, 2006). Phosphatidylcholine is pivotal for cellular function and impairment of phosphatidylcholine homeostasis is involved in many neurodegenerative disorders (Fernández-Murray and McMaster, 2005). For example, phosphatidylcholine degradation was found in individuals with senile dementia, whereas elevated phosphatidylcholine levels were found in specific brain regions of Alzheimer's patients (Klein, 2000; Esper et al., 2006). Mutations within the encoding region of the catalytic domain of NTE were shown to cause a recessively inherited spastic paraplegia in humans (Rainier et al., 2008). Furthermore, mice with a conditional deletion of NTE in neuronal cells displayed distal degeneration of the longest spinal axons (Read et al., 2009).

The neuregulin-1 family comprises more than 15 membrane-associated or secreted proteins (Falls, 2003; Esper et al., 2006). All neuregulin 1 isoforms share an epidermal growth factor (EGF)-like signaling domain, which is necessary and sufficient for the activation of ErbB receptor tyrosine kinases in oligodendrocytes and Schwann cells (Adlkofer and Lai, 2000; Falls, 2003; Esper et al., 2006; Nave and Salzer, 2006). Two isoforms of neuregulin 1, type I and type III, are thought to control migration and development of the distinct neural crest cell populations in mice. The two isoforms are expressed in distinct cell populations. Type I neuregulin 1 is produced by mesenchymal cells along the migratory route, whereas type III neuregulin 1 is produced by peripheral neurons surrounded by Schwann cells. Type III neuregulin 1 expressed by peripheral neurons plays an important role in Schwann cell development and myelination (Birchmeier, 2009; Newbern and Birchmeier, 2010).

The ErbB receptors were originally identified by their oncogenic potential (Zhang et al., 2007). Ligand binding to the extracellular domain of ErbB3 receptors promotes heterodimerization of ErbB2 and ErbB3 and activation of the intracellular tyrosine kinase domain. ErbB receptors activate various signaling cascades, such as the Ras/extracellular signal-regulated kinase 1/2 (ERK1/2) and phosphatidylinositol-3-kinase (PI3K)/Akt pathways (Newbern and Birchmeier, 2010). Previous studies have shown that ErbB2 and ErbB3 are expressed in myelinating Schwann cells in PNS and oligodendrocytes in CNS (Mei and Xiong, 2008; Newbern and Birchmeier, 2010) and ErbB1 expression was found to be absent in human and rodent primary Schwann cells (Tapinos et al., 2006). Neuregulin 1 and ErbB2/3 play important roles in mature Schwann cell functions (Cohen et al., 1992; Chen et al., 1994). Binding and activation of ErbB2 in myelinating Schwann cells led to demyelination (Tapinos et al., 2006).

A number of small molecule tyrosine kinase inhibitors have been developed including the well-known lapatinib, afatinib and AZD8931, and the emerging inhibitors AST-1306, AEE-788, CI-1033, CP-724714, CUDC-101, TAK-285, AC-480, PF-299804, and EKB-569. But none of them is ErbB2-specific inhibitor due to the conserved kinase structure in ErbB family receptors except CP-724714, which is an orally active reversible selective HER2 kinase inhibitor and has been discontinued in clinical development due to severe liver toxicity (Schroeder et al., 2014). Lapatinib (Tykerb, GW572016) is a member of the 4-anilinoquinazoline class tyrosine kinase inhibitors targeting both ErbB1 (epidermal growth factor receptor, EGFR) and ErbB2 (Johnston and Leary, 2006; Moy and Goss, 2006). Lapatinib is approved for use in combination with other anticancer agents for the treatment of the woman with ErbB2 (Her2)-positive breast cancers (Geyer et al., 2006). Lapatinib was also used in the study for brain metastases of HER2 overexpressing breast cancer in mice (Addeo and Caraglia, 2011; Taskar et al., 2012). Although ErbB1 is also the direct target of lapatinib, ErbB1 does not bind to neuregulin 1, and ErbB1 expression is missing in human and rodent primary Schwann cells (Tapinos et al., 2006). ErbB1 can form heterodimers with ErbB4, but the functional significance of ErbB4-ErbB1 heterodimers in the CNS remains unclear (Mei and Xiong, 2008).

Given the roles of the neuregulin 1 and ErbB2/3 signaling pathway in the migration, proliferation and differentiation of Schwann cells whose dysfunction is involved in Wallerian degeneration and possibly OPIDN, we sought to determine in this study whether the neuregulin 1/ErbB signaling system in the spinal cord and sciatic nerve was involved in the development of delayed neuropathy induced by TOCP.

## Materials and methods

### Animals and treatment

Adult Beijing white laying hens (12 months old and about 1.5 kg in weight) used in this study were purchased from the Fujia Center for Breeding Birds (Beijing, China). During the experiment, the temperature in the hen house was maintained at 22°C and 50% humidity with a light/dark cycle of 12 h. All animal procedures were performed in accordance with current China legislation and approved by the Animal and Medical Ethics Committee of Institute of Zoology, Chinese Academy of Sciences.

**Figure 1:**
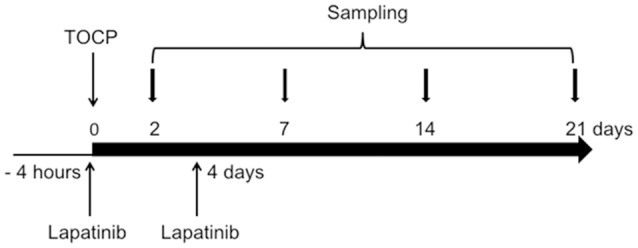
Schematic diagram for the experimental design of the *in vivo* study. The day when TOCP was administered was considered as day 0. For details, see the Materials and Methods section.

Sixty-three adult hens were divided into one control group (*n* = 7) and two treatment groups (TOCP alone and lapatinib plus TOCP; *n* = 28 per group). The birds were acclimated for at least 1 week prior to the start of the experiment. Hens in control group were administrated with vehicle alone. Hens in treatment groups were given a single dose (750 mg/kg body weight) of TOCP (purity > 99%, BDH Chemicals, London, England) in a gelatin capsule by oral gavage, which had been validated to induce OPIDN in hens (Hou et al., 2008; Xu et al., 2017) with or without pre- and post-treatment of lapatinib. Lapatinib was found to reach the peak plasma concentration at 4 h post-dosing (Paul et al., 2008). Thus, hens in lapatinib plus TOCP group were administrated with lapatinib in 10% (w/v) sulfobutyl-β-cyclodextrin by oral gavage 4 h prior to TOCP administration; and then received a second dose of lapatinib (25 mg/kg body weight, the same dosage for the two treatments) on the 4th day following TOCP administration (see Figure 1) to block the ErbB2 activation by TOCP treatment. The dose of lapatinib *in vivo* was selected based on studies in mice, which used 100 mg/kg lapatinib (Gril et al., 2008; Strecker et al., 2009; Diaz et al., 2010). With the animal equivalent dose calculation based on body surface area ratios (Nair and Jacob, 2016), lapatinib dose 100 mg/kg body weight in mice was converted into 25 mg/kg body weight in hens.

After treatment, hens were examined daily for the signs of delayed neuropathy. Hens were graded for toxicological signs of motor dysfunction on days 7, 9, 11, 13, 15, 17, 19, and 21 post-treatment by an experimenter who was blind to treatment conditions. The grade was given on a well-established 8-point scale, with 0 as normal ambulation and 8 as complete paralysis (Cavanagh et al., 1961; Pope and Padilla, 1990). For the hens which were paralyzed, water and food were made readily accessible.

On days 2, 7, 14, and 21 after TOCP administration, four hens from both treatment groups (TOCP and TOCP plus lapatinib groups) were sacrificed at each time point by cervical decapitation. Four hens in the control group were sacrificed on day 21. Brain, spinal cord, and sciatic nerves were quickly dissected and frozen in liquid nitrogen before storing at −80°C for future use in Western blotting.

For immunofluorescent staining, the other three hens from each treatment group at each time point were anesthetized by intraperitoneal injection of sodium pentobarbital (60 mg/kg body weight). Three hens in the control group were anesthetized on day 21 (see Figure 1). The hens were then perfused through the ascending aorta with 100 ml 0.9% NaCl at room temperature followed by 800 ml of 0.1 M phosphate buffer solution (PBS) (pH 7.4) buffered 4% (w/v) ice-cold paraformaldehyde for 1 h using a peristaltic pump. The perfused spinal cord and sciatic nerve tissues were postfixed in 4% paraformaldehyde for 24 h at 4°C. Then, they were stored in 0.1 M PBS buffered 30% (w/v) sucrose at 4°C for 2 days.

### Cell culture

Human Schwann cell-like sNF96.2 cells isolated from a patient with malignant peripheral nerve sheath tumor (MPNST) were obtained from ATCC (Manassas, VA, USA) and maintained in Dulbecco's modified Eagle's medium (Sigma-Aldrich Co., St. Louis, MO, USA) supplemented with 10% (v/v) fetal calf serum (Chuanye Biosciences, Tianjin, China), 100 IU/ml penicillin, and 100 μg/ml streptomycin. Incubations were carried out at 37°C in a humidified atmosphere of 5% CO_2_/95% air. The cells were seeded at a density of 1 × 10^6^ cells per 100-mm culture dish. Twenty-four hours later, the cells were subjected to various treatments. The cells were treated with vehicle, lapatinib, TOCP alone or a combination of lapatinib and TOCP for 24 h (Xu et al., 2017). Lapatinib was dissolved in DMSO and added to the cell culture medium at a concentration of 5 μM 2 h prior to the treatment of 1 mM TOCP (dissolved in DMSO) in the lapatinib plus TOCP treatment group.

### Immunofluorescence staining

The spinal cord was frozen on dry ice and cut into 15 μm coronal sections on a cryostat. The sciatic nerve was frozen on dry ice and cut into 10 μm coronal and longitudinal sections. All spinal cord and sciatic nerve sections were fixed in 4% paraformaldehyde in PBS for 10 min at room temperature (RT), washed three times with PBS, permeabilized with 0.5% (v/v) Triton X-100 in PBS for 15 min and blocked with 10% bovine serum album (BSA) in PBS for 1 h at RT. Sections were incubated for 2 h at RT with anti-β-tubulin-III (T2200) and anti-neurofilament 200 antibodies (N0142) (1:300, Sigma-Aldrich Co., St. Louis, MO, USA) followed by incubation with secondary antibodies, TRITC-labeled goat anti-rabbit IgG (CW0160) and FITC-labeled goat anti-mouse IgG (CW0512) (1:100, Cowin Biotech, Beijing, China) for 1 h. Nuclei were stained with Hoechst33258 (Sigma-Aldrich Co., St. Louis, MO, USA) for 10 min. Coverslips were mounted onto microscope slides with a fluorescence mounting medium. Four sections for each sample were stained, and six fields from each section were also randomly chosen to be counted and analyzed. The single axon could be visualized by the positive staining of neuron-specific neurofilament protein NF-200 or microtubule protein β-tubulin-III, while degenerated axons were characterized by the loss of the staining for these cytoskeleton proteins. The experimenters were blind to the treatment conditions during the counting.

Cells were cultured on coverslips and then treated with various chemicals for 24 h. Cells were subsequently fixed, permeabilized, blocked, and then incubated with the same primary and secondary antibodies as that were used for tissue sections. All the slices were visualized and photographed by Zeiss LSM 710 confocal microscopy.

### Semi-thin staining of spinal cord and sciatic nerve

Part of lumbar spinal cord and sciatic nerve (1 mm^3^) were immediately removed after being perfused and were postfixed in fixed solution [containing 4% paraformaldehyde and 2.5% (v/v) glutaraldehyde in 0.1 M phosphate buffer (pH 7.4)] for 2 h. The specimens were then treated with 1% (v/v) OsO_4_ in 0.1 M phosphate buffer (pH 7.4), dehydrated, and embedded in epoxy resin. The 200 nm semi-thin sections were stained with 1% (w/v) toluidine blue staining solution for 1 min and rinsed with distilled water. Four sections were randomly chosen for staining, and six fields from each section were also randomly counted and analyzed. The experimenters were blind to the treatment conditions during the counting. All of the slices were visualized and photographed using Carl Zeiss Microscope with an imaging software of AxioVision Rel. 4.8. The axons wrapped around by undamaged myelin sheath were considered as intact axons.

### NTE activity assay

Nervous tissues were homogenized in TE buffer (50 mM Tris-HCl, 0.2 mM ethylenediaminetetraacetic acid [EDTA], pH 8.0) and centrifuged at 100 g at 4°C for 2 min. NTE activity in the supernatant fraction was determined by colorimetric assay of phenol according to the method of Fernandes et al. (2015) and Kayyali et al. (1991) with some modifications. The NTE activity was reflected by the difference of phenol formed by phenyl valerate (synthesized in our laboratory) hydrolysis between samples exposed to 50 μM paraoxon (Sigma-Aldrich Co., St. Louis, MO, USA) and those exposed to 50 μM paraoxon plus 25 μM mipafox (synthesized in our laboratory) at 37°C. Concentration of protein was measured by the method of Bradford using BSA as the standard.

### HPLC analysis of lipids in sciatic nerve

Lipids were extracted from nervous tissues with a chloroform/methanol mixture (2:1, v/v) (Hou et al., 2008). The organic phase was evaporated under a gentle nitrogen air stream, and the extract was reconstituted in 0.5 mL of chloroform-methanol (1:1, v/v) before injected into the chromatographic system. The Agilent HPLC system consisted of an autosampler (Agilent Technologies, Santa Clara, CA, USA) and an evaporative light scattering detector (ELSD) (Agilent Technologies, Santa Clara, CA, USA). The separations were performed in a 5-μm ROBAX Silica analytical column (250 × 4.6 mm i.d.). The injection volume was 10 μL. Two mobile phases were used: (A) chloroform-methanol (7:3, v/v) and (B) chloroform-methanol-water-ammonium hydroxide (4.5:4.5:0.95:0.05, v/v/v/v). Total run time was 30 min, with a solvent flow rate of 1 ml/min. High purity nitrogen gas was used as the carrier at a flow rate of 1.5 L/min in the ELSD system, which was operated at 72°C. About 5 mg/mL standard stock solutions of phosphatidylcholine in chloroform-methanol (1:1, v/v) was stored at −20°C and protected from light. Standard solutions of phosphatidylcholine (Sigma-Aldrich Co., St. Louis, MO, USA) with concentrations 100–1000 μg/mL were prepared by serial dilution from stock solution. The limit of detection was the concentration that produced a signal to noise (S/N) ratio of 3, and the limit of quantification was defined as the lowest concentration in the calibration curve with acceptable accuracy and precision.

### Crystal violet staining

For the cell density assay, cells were seeded in 96-well plates with 5 × 10^3^ cells per well. Twenty four hours later, the cells were subjected to various treatments. The cell density in each well was determined by using the crystal violet staining method (Wang et al., 2009). The absorbance values of each well were measured at 560 and 405 nm with a multi-mode microplate reader (FLUOstar Omega, BMG Labtech, Ortenberg, Germany), and the differences in the absorbance values at these two wavelengths were used to determine the cell density.

### RT-PCR analysis

Total RNA from brain, spinal cord, and cells were extracted with TRIzol reagent (ThermoFisher Scientific, Waltham, MA, USA) according to the manufacturer's instruction. RNA was quantified by spectrophotometer after DNase I treatment. A total of 4.4 μg of each RNA sample was subjected to electrophoresis in a 1.4% agarose formaldehyde gel to verify its integrity. The total RNA (1 μg) was reverse transcribed using a first-strand cDNA synthesis kit (Toyobo Co., Osaka, Japan) in a final volume of 20 μL according to the manufacturer's instruction. The RNA from mouse and hen brain tissue was used as the positive control for NTE expression analysis. PCR analysis was then performed on aliquots of the cDNA preparations to detect NTE and GAPDH gene expression. The reactions were carried out in a volume of 25 μL containing 2.5 U of Taq DNA polymerase, 0.2 mM deoxyribonucleoside triphosphates (dNTPs), 1 × reaction buffer and 100 pmol of specific 5′ and 3′ primers. The forward primer of NTE was 5′- CCAAGAGTTCCGGCTGTCA-3′, and the reverse primer of NTE was 5′ -CA-CAATGAGGATGCAGTCGG-3′. The forward and reverse primers for the GAPDH sequence were 5′- ATGCTGGCGCTGAGTACGTC-3′ and 5′- GGTCATGAGTCCTT-CCACGATA-3′, respectively. For the indicated gene amplification, the denaturation cycle (94°C, 5 min) was followed by 35 amplification cycles. Each cycle consisted of DNA denaturation at 94°C for 30 s, primer annealing at 52°C for 30 s, and elongation at 72°C for 1 min. An elongation cycle at 72°C for 10 min was used at the end of amplification. PCR products were detected with a ChemiDoc XRS (Bio-Rad, Hercules, CA, USA) using 1% (w/v) agarose gel electrophoresis and ethidium bromide staining.

### Western blotting analysis

The homogenized tissues and cells were lyzed in modified RIPA buffer (50 mM Tris, pH 8.0, 150 mM NaCl, 1% Nonidet P-40 (v/v), 0.5% (w/v) sodium deoxycholate, 0.1% (w/v) SDS, 5 mM sodium fluoride, 1 mM sodium orthovanadate, 1 mM EDTA, 5 μg/ml of aprotinin, pepstatin, and leupeptin, 1 mM phenylmethylsulfonyl fluoride [PMSF]), and the cell lysate was incubated for 1 h on ice. Tissue and cell debris were pelleted at 12, 000 rpm at 4°C for 15 min. Protein concentration of supernatant was measured by the method of Bradford using BSA as the standard. The protein was boiled for 5 min at 100°C. Then, 20 μg of protein from each denatured sample was separated by SDS-polyacrylamide gel electrophoresis, then transferred to a polyvinylidene fluoride (PVDF) membrane (EMD Millipore, Billerica, MA, USA). Following transfer, membranes were blocked with 1 × Tris-buffered saline (TBS) buffer containing 0.1% (v/v) Tween-20 and 5% (w/v) non-fat milk for at least 1 h at room temperature, and then incubated with various primary antibodies, including rabbit anti-NTE polyclonal antibody (H-200), rabbit anti-ErbB2 (sc-284), rabbit anti- ErbB3 (sc-285), rabbit anti-neuregulin 1 (sc-348), and rabbit anti-S-100 β chain (sc-28533) antibodies, which were obtained from Santa Cruz Biotechnology (Dallas, Texas, USA); and rabbit anti-phospho-Akt (9271), rabbit anti-Akt (9272), rabbit anti-phospho-p44/42 ERK (4370), and rabbit anti-ERK (9102) antibodies, which were obtained from Cell Signaling Technologies (Danvers, MA, USA), and mouse anti-GAPDH (KC-5G4) and rabbit anti-β-actin (CW0097), which were obtained from Cowin Biotech (Beijing, China), overnight at 4°C. Then the membrane was incubated with HRP-conjugated secondary antibodies (HRP-labeled goat anti-mouse IgG [CW0102] or HRP-labeled goat anti-rabbit IgG [CW0103], which were obtained from Cowin Biotech [Beijing, China]) for 1 h at RT. Immunoreactive bands were detected using a ChemiDoc XRS system (Bio-Rad, Hercules, CA, USA). The bands then were quantitated and analyzed by Quantity One software (Bio-Rad).

### Statistical analysis

All experimental data were processed using the statistical software package SPSS, version 21.0 (SPSS Inc., Chicago, IL, USA). All data were expressed as the mean ± SEM. For the sign score analysis, statistical comparisons were performed using repeated measures two-way analysis of variance (ANOVA) with treatment as between-subject and time (day) as within-subject followed by Newman-Keuls multiple range *post-hoc* test, For the rest of comparison, we took the treatment as the only factor. So one-way ANOVA followed by Newman-Keuls multiple range *post-hoc* test was performed and results were considered statistically significant at *P* < 0.05 and extremely significant at *P* < 0.01.

## Results

### Lapatinib attenuated the signs of neuropathy induced by TOCP

**Figure 2:**
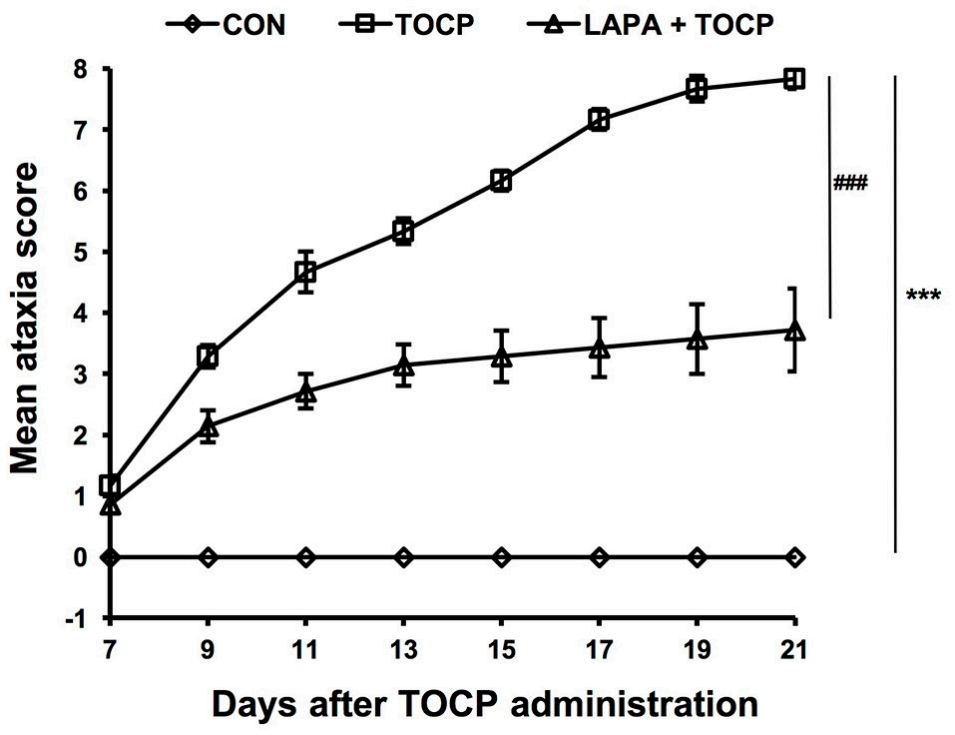
The mean ataxia scores of hens following TOCP and lapatinib treatments. After treatment, hens were observed from 7 to 21 d using an 8-point graded scale (0—no signs to 8—complete paralysis). Data were shown as Mean ± SEM. CON, control; LAPA, lapatinib. ***P < 0.001 compared with the control group. ^###^*P* < 0.001 compared to the corresponding TOCP treatment group. *n* = 7.

Hens treated by TOCP began to display slight incoordination on day 7 (Figure 2). The toxic signs aggregated over time, and hens in TOCP-treated group developed severe ataxia on day 13 post-TOCP treatment (mean score = 5.3). Most TOCP-treated hens showed total paralysis on day 17 (mean score = 7.2). Hens were unable to stand and were completely paralyzed on day 21 after TOCP dosing (mean score = 7.8). Lapatinib significantly attenuated TOCP-induced paralysis, although there was no difference in the latency to OPIDN onset between TOCP and lapatinib plus TOCP groups. The mean score of hens in lapatinib plus TOCP group was 3.1 on day 13. The final average score of hens in the lapatinib groups was 3.7 on day 21, whereas the TOCP group average was 8.0 (hens' hindlimbs were completely paralyzed). Compared with the control group, both TOCP and lapatinib plus TOCP treatment groups have significantly increased ataxia score [treatment main effect, *F*_(2, 96)_ = 969.1, *P* < 0.0001; time main effect, *F*_(7, 48)_ = 35.03, *P* < 0.0001 and treatment × time interaction, *F*_(14, 96)_ = 23.26, *P* < 0.0001]. *Post-hoc* test following ANOVA revealed a significant difference in the ataxia score among the three groups, i.e., control, TOCP and lapatinib plus TOCP treatment groups (*P* < 0.001).

### Lapatinib ameliorated the TOCP-induced axonal degeneration in spinal cord and sciatic nerve

**Figure 3:**
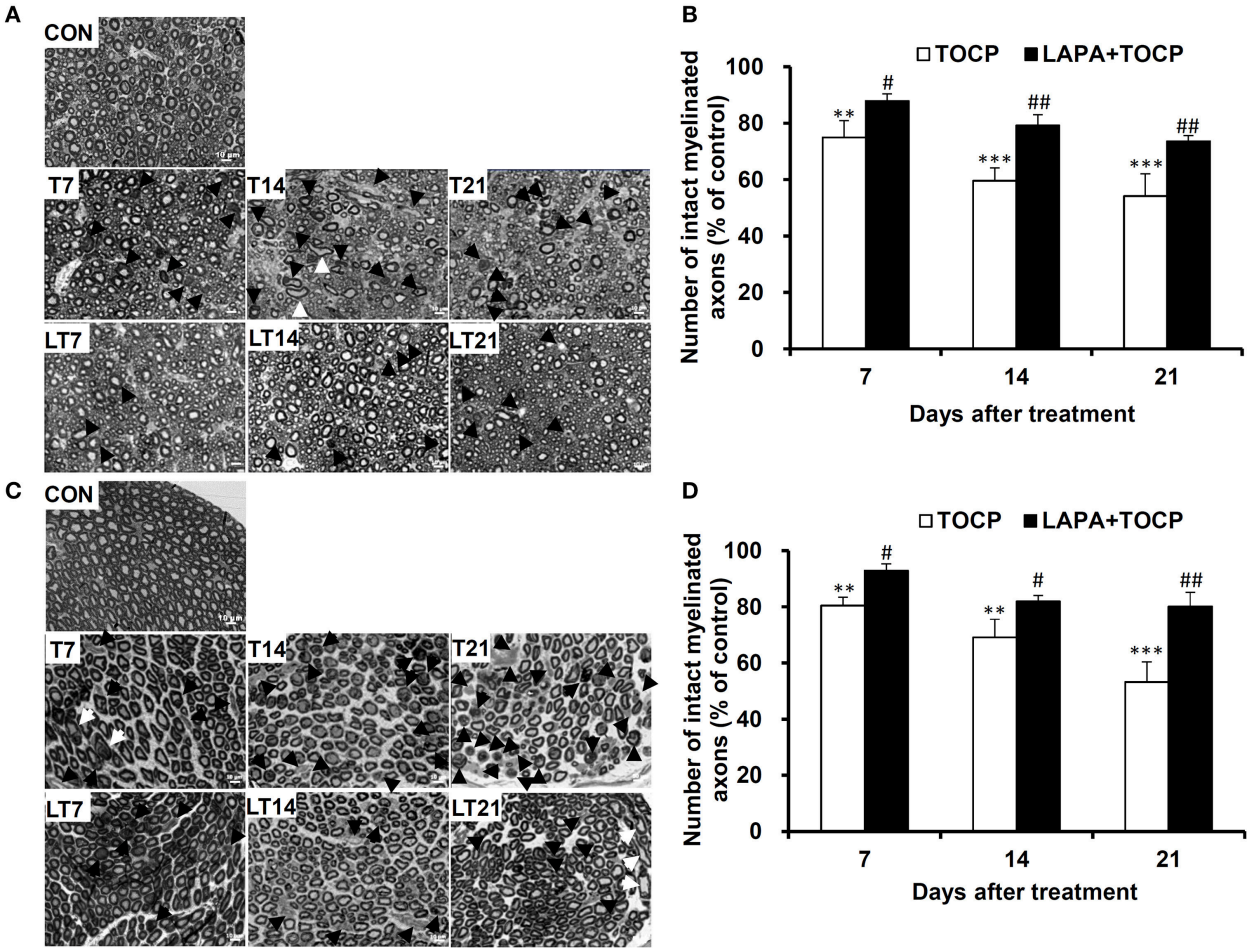
The histopathological changes following TOCP and lapatinib treatment. **(A,C)** The histopathological examination with semi-thin sections (200 nm thick) of anterior funiculus of lumbar spinal cord **(A)** and sciatic nerve **(C)**. Black arrows, white arrows, and black arrowheads indicate degenerative, swollen, and demyelinated axons, respectively. Scale bar: 10 μm. **(B,D)** The quantified numbers of intact myelinated axons in the spinal cord **(B)** and sciatic nerve **(D)**, which was shown in panels **A** and **C**, respectively. The mean numbers of intact myelinated axons in control groups of spinal cord and sciatic nerve are 5,078 ± 245 and 8,117 ± 738/mm^2^, respectively. **P < 0.01; ***P < 0.001 compared with the control group. ^#^*P* < 0.05; ^##^*P* < 0.01 compared to the corresponding TOCP treatment group.

The damage of long and large-diameter axons in the spinal cord and sciatic nerve was typical histopathological changes in OPIDN. Thus, we investigated the histopathological changes in neural fibers in the anterior funiculus of the lumbar spinal cord and sciatic nerve. Staining of semi-thin sections of spinal cord (Figure 3A) and sciatic nerve (Figure 3C) showed that, seven days after TOCP treatment, densely dyed aggregates within some axons occurred (indicated by arrows and arrowheads for degenerative and demyelinated axons, respectively, in Figure 3). On day 14 post TOCP-dosing, more axons had thinning of the myelin sheath, and even a complete loss of the myelin sheath. On day 21, some small-caliber axons regenerated and appeared in the spinal cord. The histograms displayed that the number of axons with an intact myelin sheath decreased concomitantly with the development of OPIDN (Figures 3B,D). The number drastically decreased to 54.1 and 53.2% of the control group in the spinal cord and sciatic nerve, respectively, on day 21, after TOCP dosing. However, lapatinib significantly ameliorated the axonal damage induced by TOCP exposure. In the lapatinib plus TOCP treatment group, the number of intact neural fibers reached 74.6 and 80.1% of the control group on day 21 in the spinal cord and sciatic nerve, respectively.

**Figure 4:**
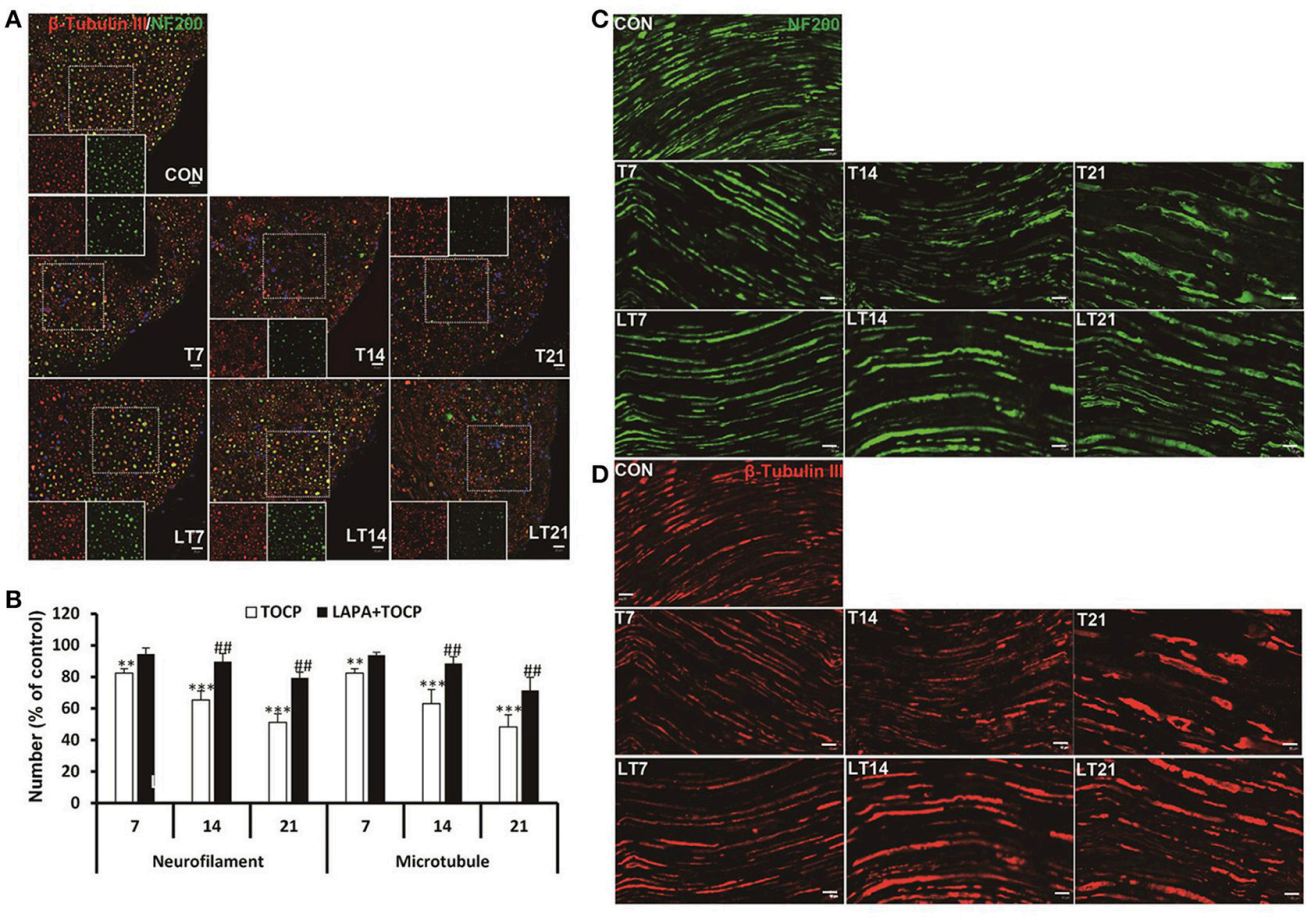
Alteration of axon numbers in spinal cord and sciatic nerve after various treatments by immunofluorescent staining of cytoskeletal proteins. **(A)** Immunofluorescent staining of spinal cord cross section for the neuron-specific microtubule protein (β-tubulin III, red) and neurofilament heavy chain (NF 200, green). Hoechst 33258 (blue) was used to stain nuclei. The single channel images of NF 200 and β-tubulin III from the region within the marked white square were shown in each treatment group. Scale bar: 20 and 5 μm outside and inside white square, respectively. **(B)** The numbers of axons expressing NF 200 and β-tubulin III, respectively, in anterior horn of lumbar spinal cord. The mean numbers of axons expressing NF 200 and β-tubulin III were 8,109 ± 254/mm^2^ and 8,926 ± 250/mm^2^ in control group, respectively (*n* = 3). **(C,D)** Immunofluorescent staining of β-tubulin III (red) and NF 200 (green) on longitudinal sections of sciatic nerve. Scale bar: 20 μm. CON, control; LAPA, lapatinib; T and LT plus number indicates the days after TOCP treatment and the treatment of lapatinib plus TOCP, respectively. **P < 0.01; ***P < 0.001 compared with the control group. ^##^*P* < 0.01 compared with the corresponding TOCP treatment group.

OPIDN features excessive degradation of cytoskeletal proteins in axons in the anterior funiculus of the lumbar spinal cord and sciatic nerve. Thus, we examined the alteration of axon numbers in spinal cord and sciatic nerve after various treatments with immunofluorescent staining of cytoskeletal proteins. Consistently, we found that the number of axons expressing neurofilament heavy chain (NF 200) and neuron-specific microtubule protein (β-tubulin III) progressively decreased with the progression of OPIDN in the anterior funiculus of the lumbar spinal cord (Figure 4A). The numbers of axons expressing NF 200 and β-tubulin III were 51.2 and 48.2% of that in control group on day 21 after TOCP dosing, respectively (*P* < 0.001) (Figure 4B). However, in the lapatinib plus TOCP treatment group, the number of axons expressing NF 200 and β-tubulin III reached 79.4 and 71.3% of that of the control group, respectively, on day 21 after TOCP administration (*P* < 0.01 compared with TOCP group).

Immunofluorescence staining of longitudinal sections of sciatic nerve showed that most axons had intact structures in the control treatment group (Figures 4C,D). However, more axons showed fragmented staining from day 7 to day 21. On the contrary, no notable degeneration of axons was seen in the lapatinib plus TOCP treatment group, which suggested that lapatinib prevented the axonal degeneration in OPIDN induced by TOCP.

### Lapatinib maintained NTE activity and phosphatidylcholine homoeostasis in the sciatic nerve

We then determined the effects of TOCP and lapatinib on NTE activity and phosphatidylcholine levels in spinal cord and sciatic nerve. The results for the inhibition of NTE activity by TOCP in spinal cord and sciatic nerve were consistent with our previous study (Hou et al., 2008). As shown in Table 1, both TOCP and lapatinib plus TOCP treatment significantly inhibited the NTE activity at all time points in spinal cord and sciatic nerve, and no significant difference was detected between the two groups in the spinal cord. Interestingly, in the sciatic nerve, NTE activity in the lapatinib plus TOCP treatment group was significantly greater than the corresponding TOCP alone treatment group on the 7th day (*P* < 0.01).

**Table 1:** The activity of NTE (nmol phenol/min/mg protein) after indicated treatments in hen's nervous tissues.

**Tissue**	**Sampling time (days)**	**TOCP**	**Lapatinib + TOCP**
Spinal cord	2	0.93 ± 0.25 (91)	1.15 ± 0.36(88)
	7	4.06 ± 0.22 (59)	3.43 ± 0.17(65)
	14	6.04 ± 0.35 (39)	6.69 ± 0.45(33)
	21	9.74 ± 0.33 (2)	8.83 ± 0.83(11)
Sciatic nerve	2	0.51 ± 0.09 (81)	0.50 ± 0.09(82)
	7	1.37 ± 0.24 (50)	1.98 ± 0.59(28)^##^
	14	2.04 ± 0.36 (26)	1.90 ± 0.19(31)
	21	2.21 ± 0.13 (20)	2.22 ± 0.54(20)

Data were expressed as mean ± SE (n = 4). NTE activity in control spinal cord and sciatic nerve was 9.94 ± 0.55 and 2.75 ± 0.41 nmol phenol/min/mg protein, respectively. The numbers in parentheses represent the percentage of inhibition (%).

## *P < 0.01 compared with the corresponding TOCP treatment group*.

**Figure 5:**
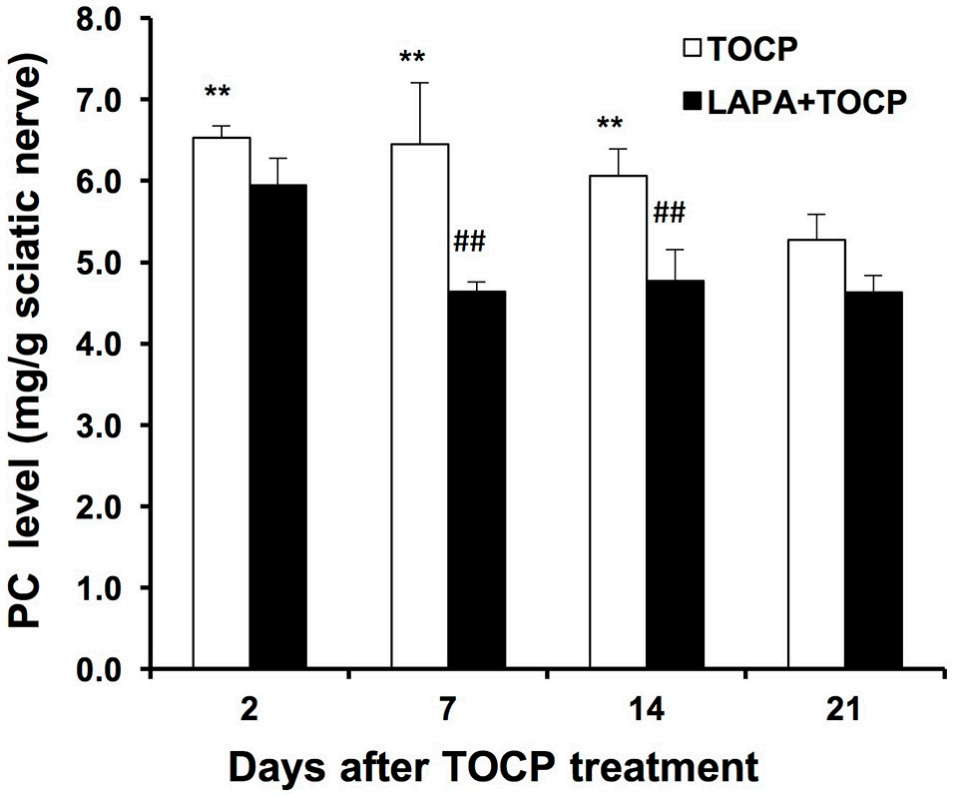
Phosphatidylcholine level (mg/g tissue) in sciatic nerve of hens dosed with TOCP or TOCP plus lapatinib. Data were expressed as mean ± SE (*n* = 4). The phosphatidylcholine (PC) level in the sciatic nerves from the control hens was 4.54 ± 0.16 mg/g tissue. **P < 0.01 compared with the control group; ^##^*P* < 0.01 compared with the corresponding TOCP treatment group.

We subsequently monitored whether the inhibition of NTE activity could affect the level of its substrate, phosphatidylcholine, in the sciatic nerve. The level of phosphatidylcholine increased from the 2nd day after TOCP dosing (43.8% increase compared with that in the control group, *P* < 0.01). Phosphatidylcholine did not reach basal levels until the day 21 after TOCP treatment (Figure 5). There was no significant difference in phosphatidylcholine level between TOCP and lapatinib plus TOCP groups (*P* > 0.05) on the 2nd day. However, phosphatidylcholine level in the lapatinib plus TOCP treatment groups was significantly lower than that in the TOCP alone treatment group from the day 7 to day 14 (28.2% and 21.3% decrease, respectively, *P* < 0.01), and phosphatidylcholine level in the lapatinib plus TOCP treatment group decreased rapidly to basal levels on the day 7 after TOCP administration. In spinal cord, there was no significant difference of the PC level between TOCP and lapatinib plus TOCP group at any time-point (data not shown).

### Lapatinib inhibited neuregulin 1/ErbB signaling pathway activated by TOCP in spinal cord and sciatic nerve

**Figure 6:**
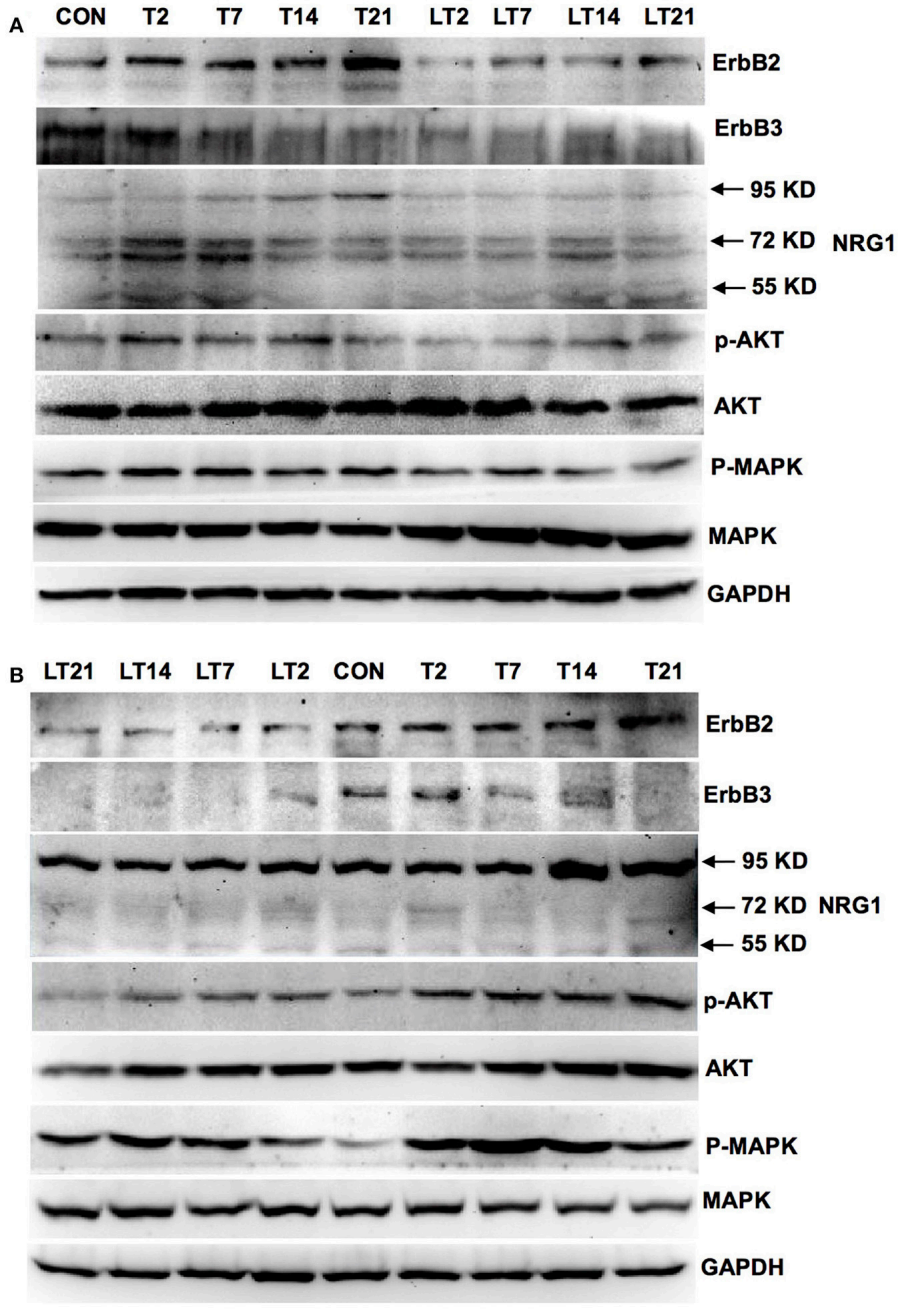
Activation of neuregulin 1/ErbB signaling pathway in spinal cord and sciatic nerve of adult hens after TOCP treatment. Protein expression levels in spinal cord **(A)** and sciatic nerve **(B)** were measured by Western blotting analysis, respectively. CON, control; T and LT plus number indicated the days after TOCP treatment and lapatinib combined with TOCP treatment, respectively. GAPDH was used as the loading control.

We investigated whether neuregulin 1/ErbB2 and its downstream signals were activated in hens exposed to TOCP or lapatinib plus TOCP. In the spinal cord (Figure 6A; for quantitative data, see Table 2), ErbB2 expression was induced by TOCP treatment and inhibited by lapatinib to levels even below that of the control group (LT2-LT21). However, expression of ErbB3 decreased gradually following TOCP treatment, and lapatinib accelerated the decrease of ErbB3 expression. Neuregulin 1 levels were induced by TOCP, and the increase in neuregulin 1 levels was blocked by lapatinib. TOCP stimulated mild activation of Akt (p-Akt) and ERK (p-ERK), whereas lapatinib blocked TOCP-induced Akt and ERK activation.

**Table 2:** The quantitative analysis of neuregulin 1 (NRG1)/ErbB signaling molecules in spinal cord and sciatic nerve following various treatments.

**Tissue**	**Target proteins**	**T2**	**T7**	**T14**	**T21**	**LT2**	**LT7**	**LT14**	**LT21**
Spinal Cord	ErbB2	1.93 ± 0.24^**^	1.78 ± 0.19^**^	1.64 ± 0.17^*^	3.77 ± 0.43^**^	0.32 ± 0.04^*^^##^	0.49 ± 0.06^*^^##^	0.49 ± 0.09^##^	0.83 ± 0.08^##^
	ErbB3	0.85 ± 0.08	0.51 ± 0.04^**^	0.42 ± 0.03^**^	0.47 ± 0.06^**^	0.39 ± 0.02^**^^##^	0.31 ± 0.01^**^^#^	0.30 ± 0.04^**^	0.28 ± 0.02^**^^#^
	NRG1 (55 -72 kDa)	2.86 ± 0.05^**^	2.74 ± 0.04^**^	1.68 ± 0.10^**^	1.83 ± 0.10^**^	1.61 ± 0.02^**^^##^	1.24 ± 0.06^##^	1.69 ± 0.18^**^	1.49 ± 0.07^**^^#^
	NRG1 (72 kDa)	2.86 ± 0.08^**^	2.62 ± 0.06^**^	1.66 ± 0.09^**^	1.84 ± 0.09^**^	1.62 ± 0.03^**^^##^	1.08 ± 0.03^##^	1.19 ± 0.12^##^	1.24 ± 0.06^##^
	NRG1 (95 kDa)	0.88 ± 0.02	1.51 ± 0.14^**^	2.01 ± 0.12^**^	2.80 ± 0.04^**^	0.92 ± 0.07	0.77 ± 0.06^##^	0.95 ± 0.04^##^	1.12 ± 0.12^##^
	p-AKT/AKT	1.51 ± 0.11^*^	1.39 ± 0.14^*^	1.54 ± 0.1^**^	1.35 ± 0.14	0.81 ± 0.08^##^	0.88 ± 0.05^##^	1.04 ± 0.18^##^	1.04 ± 0.06
	p-MAPK/MAPK	1.93 ± 0.09^**^	1.76 ± 0.10^**^	1.42 ± 0.12^*^	1.41 ± 0.03^*^	0.91 ± 0.10^##^	0.83 ± 0.10^##^	0.77 ± 0.04^##^	0.78 ± 0.04^##^
Sciatic Nerve	ErbB2	1.46 ± 0.05^**^	1.50 ± 0.09^**^	1.72 ± 0.13^**^	2.20 ± 0.17^**^	0.61 ± 0.08^**^^##^	0.42 ± 0.09^**^^##^	0.44 ± 0.05^**^^##^	0.32 ± 0.03^**^^##^
	ErbB3	1.12 ± 0.10^**^	0.89 ± 0.05^**^	0.70 ± 0.08^**^	0.16 ± 0.05^**^	0.47 ± 0.07^**^^##^	0.17 ± 0.02^**^^#^	0.20 ± 0.06^**^	0.12 ± 0.03^**^^#^
	NRG1 (95 kDa)	1.02 ± 0.08	0.98 ± 0.07	1.71 ± 0.12^**^	1.89 ± 0.08^**^	1.01 ± 0.09	0.95 ± 0.04	1.02 ± 0.04^##^	1.03 ± 0.04^##^
	p-AKT/AKT	2.62 ± 0.15^**^	2.35 ± 0.16^**^	2.05 ± 0.15^**^	2.47 ± 0.18^**^	1.23 ± 0.12^##^	1.28 ± 0.21^##^	1.10 ± 0.10^##^	0.76 ± 0.05^##^
	p-MAPK/MAPK	3.00 ± 0.09^**^	4.11 ± 0.10^**^	3.25 ± 0.16^**^	3.10 ± 0.22^**^	1.15 ± 0.10^##^	2.24 ± 0.11^**^^##^	1.97 ± 0.15^**^^##^	1.48 ± 0.10^*^^##^

Data were expressed as mean ± SE (n = 4). Each band was normalized with house-keeping protein GAPDH and expressed as the fold change of control.

* P < 0.05;

** P < 0.01 compared with the control group.

# P < 0.05;

## *P < 0.01 compared with the corresponding TOCP treatment group. T and LT plus number indicate the days after TOCP and lapatinib plus TOCP treatments, respectively*.

**Figure 7:**
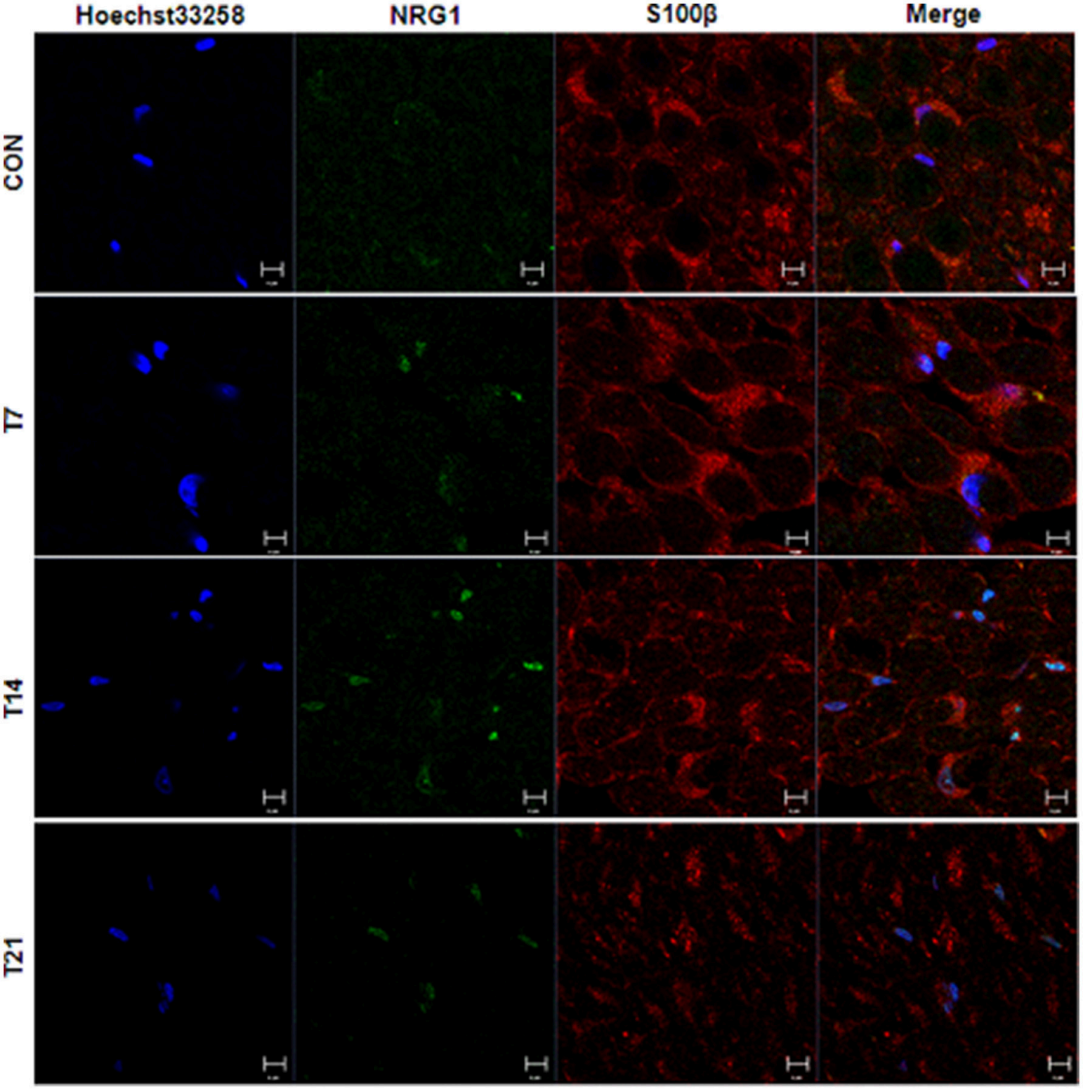
Immunofluorescence staining of neuregulin 1 and Schwann cell specific S100β in sciatic nerve of hens treated with TOCP. Adult hens were administrated with TOCP (750 mg/kg body weight, po), and then the sciatic nerve samples were collected on days 7, 14, and 21. Anti-neuregulin 1 (sc-348) (green) and anti-S-100 β chain (sc-28533) (red) were used to detect neuregulin 1 and label Schwann cells, respectively. Hoechst 33258 (blue) was used to mark nucleus. The histogram in lower panel was the quantitative result of the immunofluorescent staining. CON, control; T plus number indicated the sampling time (days) after TOCP treatment. Scale bar: 5 μm.

In the sciatic nerve (Figure 6B; for quantitative data, see Table 2), the general pattern of protein expression was similar to that of the spinal cord. ErbB2 expression increased on the 2nd, 7th, and 14th day after TOCP dosing compared to that of the control group. ErbB2 expression was prominently induced on day 21 in TOCP group. Lapatinib evidently inhibited the ErbB2 expression induced by TOCP. Neuregulin 1 expression was induced on the 2nd and 7th day after TOCP dosing in the sciatic nerve, and lapatinib blocked TOCP-induced neuregulin 1 expression. Furthermore, our fluorescent staining for neuregulin 1 expression in the sciatic nerves revealed that TOCP induced neuregulin 1 expression in both nuclei and cytoplasm of Schwann cells of the sciatic nerves (Figure 7). ErbB3 expression decreased progressively from the 2nd day, whereas lapatinib downregulated ErbB3 expression from the 7th to 21st day after TOCP dosing. The activation of Akt and ERK after TOCP treatment was more evident in the sciatic nerve than the spinal cord. Lapatinib treatment markedly inhibited Akt activation by TOCP treatment. The inhibition of ERK activation by lapatinib was evident at the 2nd and 7th day post-TOCP treatment.

### Lapatinib prevented the loss of processes induced by TOCP in sNF96.2 cell

**Figure 8:**
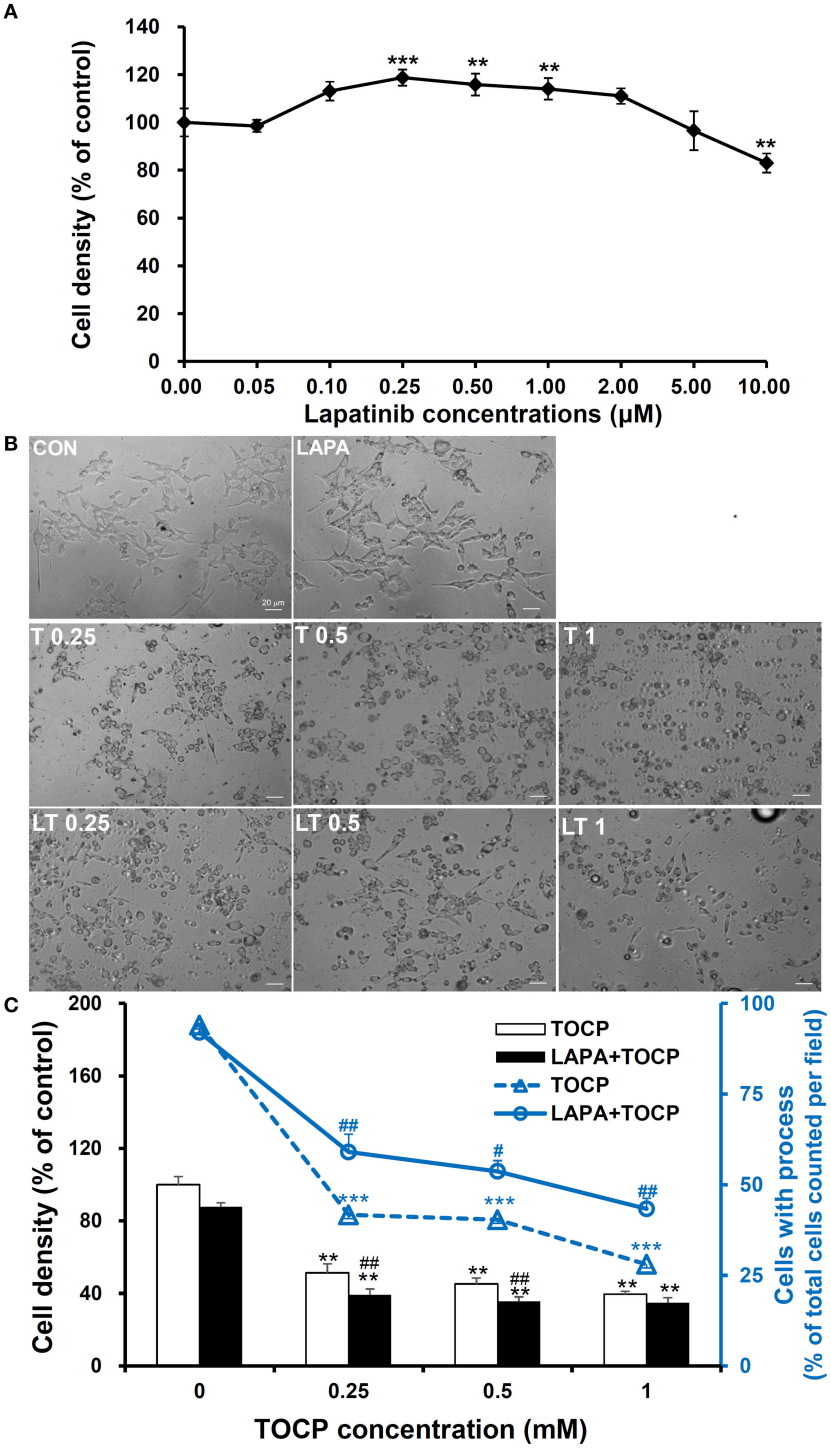
Cell density and spreading of sNF96.2 cells after TOCP and lapatinib treatment. **(A,B)** The sNF96.2 cells were exposed to various concentrations of lapatinib **(A)** or different concentrations of TOCP in presence and absence of 5 μM lapatinib **(B)** for 24 h, which was added to the medium 2 h prior to TOCP treatment. The cell density **(A)** was determined by the crystal violet staining method. Cells with prominent spindle processes in photomicrographs **(B)** were considered as spreading well. **(C)** The cell density (scaled by left vertical axis) and quantified counts (scaled by right vertical axis) of the cells with processes in panel **(B)** were determined. The y-axis of the histogram was the percentage of cells with processes in total cell number per observed field. ^**^P < 0.01; ^***^P < 0.001 compared with the control group (vehicle treatment); ^#^*P* < 0.05 and ^##^*P* < 0.01 compared with the corresponding TOCP treatment group. CON, control; LAPA, lapatinib; T and LT plus number indicated the TOCP concentration in TOCP individual treatment and TOCP combined with lapatinib treatment groups, respectively. Scale bar: 20 μm.

We used human derived sNF96.2 Schwann cell line as our *in vitro* model to look further investigate the cellular mechanisms of the effects of lapatinib on TOCP-induced neuropathy. The concentration of lapatinib used in this study was determined by analysis of cell density under with lapatinib treatments. As shown in Figure 8A, the cell density was significantly increased by treatment with lapatinib under the concentration of 0.25, 0.5, and 1.0 μM for 24 h, while the cell density decreased to control levels when the lapatinib concentration was increased to 5.0 μM. The cell density was inhibited markedly with 10 μM lapatinib treatment. Therefore, a lapatinib concentration of 5 μM was used in further cell studies. Such a concentration may allow lapatinib to maximally exert its inhibitory function without producing significant cell toxicity.

As shown in Figure 8B, TOCP significantly inhibited the cell density at the lowest concentration used (inhibition ratio > 50%, *P* < 0.01 compared with that of control group). The combination of lapatinib and TOCP also inhibited cell growth (*P* < 0.01 compared with control). The number of spreading cells with processes declined as the TOCP concentrations increased (Figures 8B,C). Lapatinib itself had no effect on cell spreading. However, lapatinib evidently attenuated the loss of processes induced by various TOCP concentrations.

### Lapatinib partially rescued NTE activity inhibited by TOCP in sNF96.2 cells

**Figure 9:**
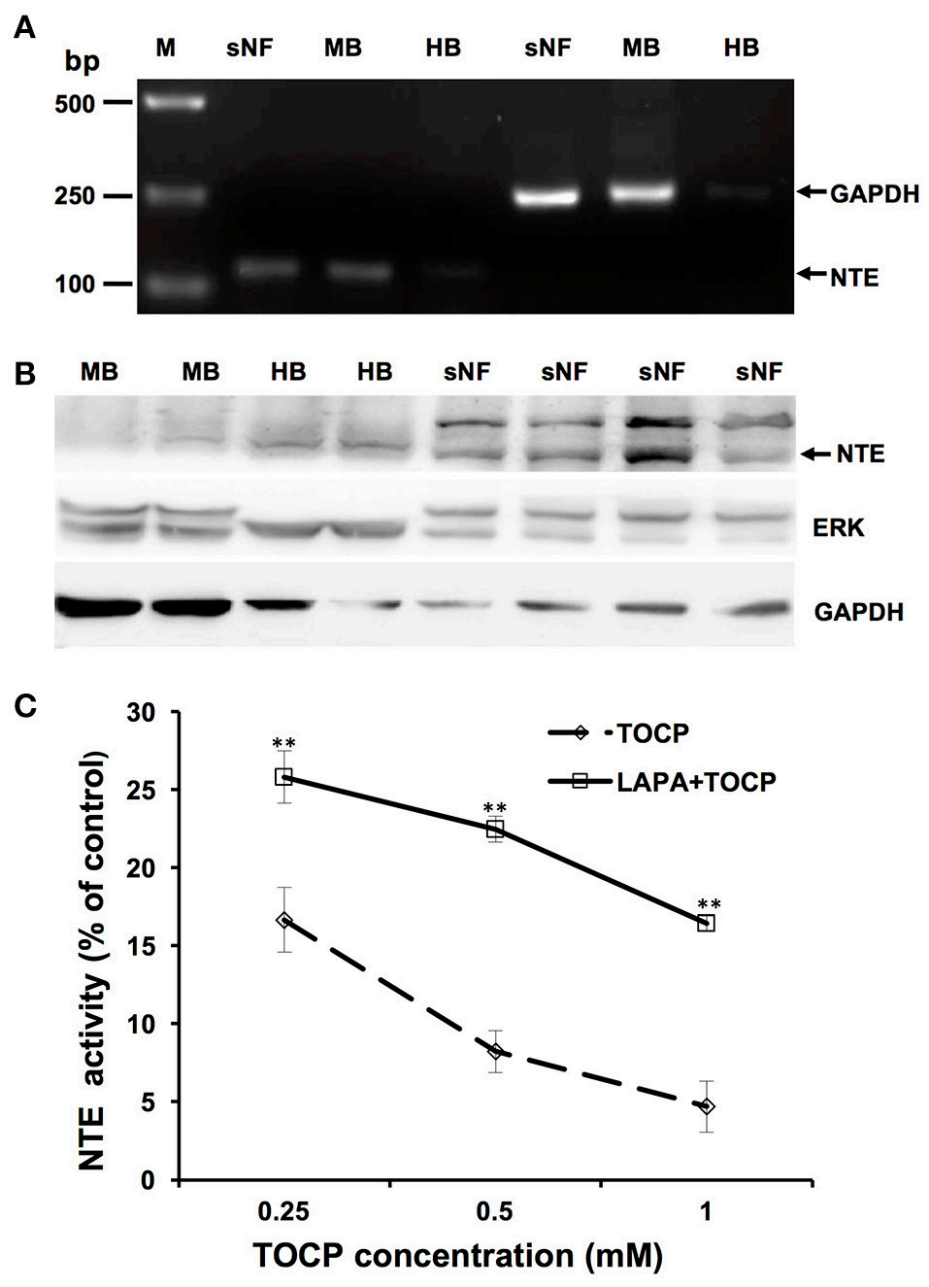
Expression and activity of NTE in sNF96.2 cells. **(A)** RT-PCR analysis of NTE in sNF96.2 cell (sNF). GAPDH was used as the loading control. **(B)** Protein expression of NTE in sNF96.2 cells. GAPDH and ERK were used as loading controls. Chemical-naive adult mouse brain (MB) and hen brain (HB) were used as positive controls for NTE expression analysis. M, Marker. **(C)** The effects of different treatments on NTE activity in sNF96.2 cells. The NTE activity in the control group was 12.04 ± 0.86 nmole phenol/min/mg protein. **P < 0.01 compared with the corresponding TOCP treatment group.

As shown in Figure 9A, the result of RT-PCR indicated sNF96.2 cells expressed NTE. Brain samples from chemical-naive adult hens and adult mice were used as positive control and a band with identical size was detected in the lane of sNF96.2 cells and positive controls. In addition, the results of the Western blotting analysis confirmed NTE protein expression in sNF96.2 cells (Figure 9B). Figure 9C showed that a relatively low concentration of TOCP (0.25 mM) significantly inhibited NTE activity (84.3% inhibition compared with control group, *P* < 0.01). Higher concentration of TOCP (1 mM) inhibited NTE activity by 95.3% compared to that of the control group (*P* < 0.01). Lapatinib by itself at the concentration of 5 μM had no effect on NTE activity (data not shown). Although lapatinib failed to rescue NTE activity to basal levels, it significantly restored NTE activity inhibited by TOCP.

### Lapatinib inhibited neuregulin 1/ErbB signaling pathway activated by TOCP in sNF96.2 cells

**Figure 10:**
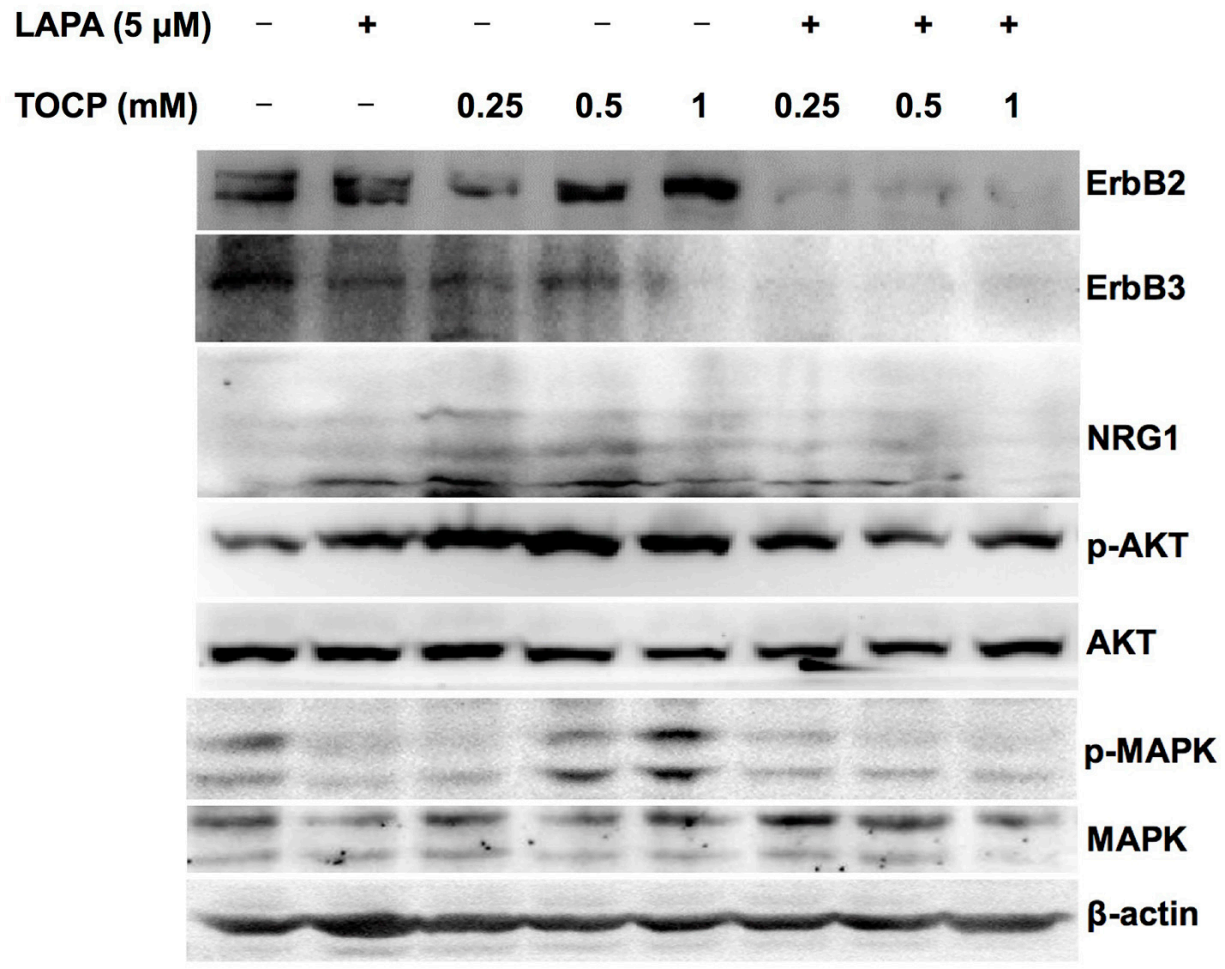
Effects of lapatinib combined with TOCP on neuregulin 1/ErbB signaling in sNF96.2 cells. Human Schwann cell line sNF96.2 cells were subjected to the indicated treatments for 24 h. β-actin was used as the loading control.

Based on the *in vivo* results, we attempted to see whether the effects of lapatinib and TOCP were direct on Schwann cells using sNF96.2 cells as the cell model. As shown in Figure 10 (for quantitative data, see Table 3), TOCP treatment led to increased expression of ErbB2, neuregulin 1, and p-Akt. An intermediate (0.5 mM) and high dose (1 mM) of TOCP induced ERK phosphorylation, while lapatinib apparently inhibited TOCP-induced activation of the neuregulin 1/ErbB signaling system.

**Table 3:** The quantitative analysis of neuregulin 1/ErbB signaling molecules in sNF96.2 cells following various treatments.

**Target Protein**	**LAPA**	**T0.25**	**T0.5**	**T1.0**	**LT0.25**	**LT0.5**	**LT1.0**
ErbB2	0.90 ± 0.10^**^	1.50 ± 0.13^**^	1.48 ± 0.08^**^	1.71 ± 0.07^**^	1.02 ± 0.07^**^^##^	0.99 ± 0.10^**^^##^	1.07 ± 0.05^**^^##^
ErbB3	0.59 ± 0.03^**^	0.52 ± 0.05^**^	0.47 ± 0.04^**^	0.40 ± 0.06^**^	0.25 ± 0.02^**^^##^	0.23 ± 0.02^**^^##^	0.24 ± 0.03^**^^#^
Neuregulin 1 (95 kDa)	1.02 ± 0.09	1.38 ± 0.06^**^	1.20 ± 0.05^*^	1.22 ± 0.04^*^	0.99 ± 0.04^**^	1.03 ± 0.03	0.34 ± 0.06^**^^##^
p-AKT/AKT	1.05 ± 0.08^**^	1.38 ± 0.04^**^	1.33 ± 0.05^**^	1.30 ± 0.09^**^	1.09 ± 0.07^##^	0.59 ± 0.10^**^^##^	0.46 ± 0.04^##^
p-MAPK/MAPK (42kDa)	0.48 ± 0.02^**^	0.59 ± 0.03^*^	1.28 ± 0.08^**^	1.62 ± 0.10^**^	0.49 ± 0.05^**^	0.42 ± 0.04^**^^##^	0.31 ± 0.04^**^^##^
p-MAPK/MAPK (44 kDa)	0.53 ± 0.04^**^	0.62 ± 0.03^*^	1.32 ± 0.03^**^	1.65 ± 0.04^**^	0.50 ± 0.03^**^	0.52 ± 0.03^**^^##^	0.49 ± 0.03^**^^##^

Data were expressed as mean ± SE (n = 3). Each band was normalized with house-keeping protein β-actin and expressed as the fold change of control.

* P < 0.05;

** P < 0.01 compared with the control group.

# P < 0.05;

## *P < 0.01 compared with the corresponding TOCP treatment group. T and LT plus number indicate different TOCP concentrations. LAPA, lapatinib; T, TOCP*.

## Discussion

The present study showed that, although the ErbB1/2 inhibitor lapatinib failed to delay the latent period of OPIDN, it effectively attenuated the neurodegenerative manifestations induced by TOCP in hens and attenuated their pathological damage in spinal cord and sciatic nerves. Previous studies have shown that the ErbB2 signaling pathway plays important roles in myelin sheath maintenance in adult vertebrates. The ErbB signaling pathway was activated in the sciatic nerve of rats with Wallerian degeneration (Guertin et al., 2005; Liang et al., 2012). PKI166, an inhibitor of ErbB1 and ErbB2 activation, drastically improved the demyelination of sciatic nerve induced by leprosy bacteria (Tapinos et al., 2006). However, PKI166 was discontinued due to severe side effects. In this current study, we chose lapatinib as the inhibitor of ErbB2 receptor, because lapatinib was approved by US Food and Drug Administration in 2007 for patients with metastatic breast cancer as it shows an exciting therapeutic effect on ErB2-positive breast cancer (Paul et al., 2008). Furthermore, we explored whether lapatinib was a useful therapeutic intervention for OPIDN when given after TOCP treatment. We treated hens with one single dose (25 mg/kg body weight) of lapatinib on the 7th day following TOCP (750 mg/kg body weight) exposure. However, the lapatinib failed to attenuate the toxic signs of OPIDN (data not shown). Thus, the propoer dose schedule of lapatinib needs to be further optimized before it can be used therapeutically to treat OPIDN.

NTE plays fundamental roles in the development and progression of OPIDN. The present results confirmed that maximum inhibition of NTE activity occurred on the 2nd day after TOCP exposure, and NTE activity was progressively restored in the spinal cord and sciatic nerve, which is consistent with the finding that the phosphatidylcholine level in the TOCP treatment group increased as early as the 2nd day after TOCP exposure (Figure 5) because of the inhibition of NTE activity by TOCP. In this study, we found that lapatinib accelerated the restoration of NTE activity in sciatic nerve on day 7, but not in spinal cord (Table 1). Notably, lapatinib restored some of NTE activity inhibited by TOCP in sNF96.2 Schwann cells (Figure 9). Based on both *in vivo* and *in vitro* results, we therefore infer that the neuregulin 1/ErbB signaling system plays important roles in the regulation of NTE activity. However, the exact relationship between neuregulin 1/ErbB system and NTE activity remains to be elucidated. Our finding that lapatinib preserved NTE activity in sciatic nerve, but not in spinal cord is consistent with the previous report showing that neuregulin 1/ErbB signaling pathway has distinct functions in central nervous system (CNS) and peripheral nervous system (PNS) (Brinkmann et al., 2008). These results suggested that neuregulin 1/ErbB may not be solely responsible for OPIDN in spinal cord, but may be the primary mechanism of OPIDN in sciatic nerve.

In addition, we found that the induction of the p-ERK and p-Akt after TOCP treatment in sciatic nerve was more evident than that in spinal cord (Figure 6), which again implied the distinct actions of TOCP to neuregulin 1/ErbB system between CNS and PNS. Activated ERK signaling was shown to inhibit remyelination and induce the dedifferentiation and proliferation of Schwann cells (Harrisingh et al., 2004; Ogata et al., 2004). The effect of axons in promoting myelination was interrupted when PI3K/Akt was blocked (Maurel and Salzer, 2000). Expression of the dominant negative mutant of PI3K or Akt in cultured Schwann cells also inhibited the myelination. Thus, the equilibrium between activated Akt and ERK was proposed to determine the differentiation of Schwann cells (Ogata et al., 2006). In the present study, both Akt and ERK signals were persistently activated after TOCP exposure. Thus, the demyelination elicited by hyperactive ERK probably played a much more important role in OPIDN than the remyelination by activated Akt.

Furthermore, we have noted in the current study that the expression of both neuregulin 1 and ErbB was induced after TOCP exposure in Schwann cells of sciatic nerve as well as in sNF96.2 Schwann cells. Thus, there may be a common regulation pathway for both ErbB and neuregulin 1 expression in Schwann cells. Therefore, lapatinib could affect not only ErbB but also neuregulin 1 expression (Figure 6). Notably, on the contrary to Wallerian degeneration, activated NRG1/ErbB signal pathway by TOCP may mediate dedifferentiation of the cells. However, detailed analysis of how ErbB activation lead to dedifferentiation of Schwann cells in OPIDN needs to be carried out in the future.

To further investigate the roles of neuregulin 1/ErbB signaling in TOCP-induced delayed neuropathy, the sNF96.2 cells were used as an *in vitro* model for Schwann cells. The present results showed that lapatinib protected cells from inhibition of cell spreading by TOCP to a large extent (Figure 8). This suggests that neuregulin 1/ErbB signaling may mediate some of the cytotoxicity of TOCP. Consistent with the *in vivo* results, TOCP treatment activated the expression of neuregulin 1/ErbB, as well as its downstream signaling molecules in sNF96.2 cells, whereas lapatinib inhibited the TOCP-induced activation of the neuregulin 1/ErbB system. In addition, we detected NTE expression at both transcriptional and translational levels in sNF96.2 cells (Figure 9). The activity of NTE in basal conditions was twice that of SH-SY5Y neuroblastoma cells (data not shown). This suggests that the sNF96.2 cell line may be a useful *in vitro* Schwann cell model for studying the mechanism of OPIDN. This is the first report to use sNF96.2 cells as an *in vitro* model in the research of axonopathies and in particular OPIDN. This cell line may be useful in subsequent studies on the role of Schwann cells in other peripheral axonopathy, such as diabetic peripheral neuropathy. Based on the current findings, it is evident that lapatinib significantly blocks the development of OPIDN. However, the question remains as to whether any direct or indirect interaction exists between TOCP and ErbB2/3. Radiolabeled TOCP may be used in future research to address this question.

## Conclusions

In summary, we found that the TOCP-induced hyperactivation of neuregulin 1/ErbB signaling in Schwann cells might lead to the disturbance of NTE activity and degenerative pathology in spinal cord and sciatic nerve. Moreover, lapatinib prevented the TOCP-induced neuropathy through attenuating the activation of neuregulin 1/ErbB signaling. These results suggest that the neuregulin 1/ErbB may play important roles in the development of OPIDN, and this signaling pathway may serve as a novel target for the treatment of OPIDN.

## Author contributions

H-YX and Y-JW: designed the research; H-YX, Y-JS, M-YX, and LZ: performed the research; H-YX, PW, and Y-JW: interpreted the data; H-YX, PW, and Y-JW: co-wrote the manuscript.

### Conflict of interest statement

The authors declare that the research was conducted in the absence of any commercial or financial relationships that could be construed as a potential conflict of interest.

## Abbreviations

BSA: bovine serum album
dNTP: deoxyribonucleoside triphosphate
EDTA: ethylenediaminetetraacetic acid
EGF: epidermal growth factor
ELSD: evaporative light scattering detector
ErbB: epidermal growth factor receptor
ERK: extracellular signal-regulated kinase
LAPA: lapatinib
NTE: neuropathy target esterase
OP: organophosphorus compounds
OPIDN: organophosphorus compound-induced delayed neuropathy
PBS: phosphate buffer solution
PI3K: phosphatidylinositol-3-kinase
PMSF: phenylmethylsulfonyl fluoride
PVDF: polyvinylidene fluoride
RT: room temperature
TBS: Tris-buffered saline
TOCP: tri-*o*-cresyl phosphate.
